# Case report: The power of immunotherapy in advanced cutaneous squamous cell carcinoma

**DOI:** 10.3389/fonc.2022.1081118

**Published:** 2023-01-04

**Authors:** Maximilian Brockwell, Marium Husain, Claire Verschraegen, Richard Wu, Gabriel Tinoco

**Affiliations:** ^1^ The Ohio State University, Columbus, OH, United States; ^2^ Division of Medical Oncology, Comprehensive Cancer Center, The Ohio State University, Columbus, OH, United States

**Keywords:** skin cancer, oncology, head and neck cancer, chemotherapy, radiotherapy, cancer intervention

## Abstract

We describe the case of a neglected cutaneous squamous cell carcinoma with extensive facial involvement. The patient is a male in his late 70s who presented to dermatology with a large destructive facial mass that had increased in size gradually over 3 years and then rapidly proliferated, consuming a large portion of his left maxillofacial region. While the immediate reaction was referral to hospice care, medical oncology recommended treatment with cemiplimab, an immune checkpoint inhibitor. Collaboration with multiple providers facilitated the delivery of a multidisciplinary approach utilizing immunotherapy with QUAD shot radiotherapy. The immunotherapy treatment resulted in a dramatic disease regression, but the large facial anatomical defect caused by the carcinoma remained. The patient is undergoing reconstructive surgeries. This case illustrates the potential for significant response with immune checkpoint inhibitors delivered in combination with cyclical hypofractionated radiation therapy for patients with cutaneous squamous cell carcinoma, even in very advanced disease.

## Introduction

Immune checkpoint inhibitors (ICIs) have revolutionized the management of different cancer types, initially melanoma and non-small cell lung cancer (NSCLC). Lately, the standard of care for advanced cutaneous squamous cell carcinoma (cSCC) has become ICIs. Until the advent of immunotherapies, patients with locally advanced cancers or distant metastases presented as a treatment challenge and had a poor prognosis (1, 2). The second most common skin cancer after basal cell carcinoma (BCC), cSCC commonly develops after long-term exposure to ultraviolet (UV) light or in response to other factors, such as an immunocompromised host, organ transplant status, familial syndromes, prior antineoplastic therapies, or other environmental exposures (for example, chemicals or burns) (3–7).

In early-stage disease, surgery remains the mainstay curative treatment. Adjuvant radiation therapy has not been well-studied in cSCC, but is typically utilized when surgical margins are unclear or if there is a high risk of local recurrence (8, 9). Radiation therapy is more beneficial in cSCC with parotid metastases than superficial parotidectomy alone (10). NCCN head and neck cancer guidelines recommend definitive radiation dosings to tumors with a diameter smaller than 2 cm or greater than 2 cm, T3/T4, or those with invasion of the bone that are inoperable (11). Palliative QUAD shot radiotherapy has been used for incurable head and neck cancer with positive results (28). Combination systemic treatment with radiation is also recommended, with guidelines recommending concurrent cisplatin and radiation or a clinical trial.

If radiation or surgery is not feasible, single-agent cemiplimab or pembrolizumab are feasible options (11). Today, cemiplimab and pembrolizumab are both FDA-approved as ICIs for the treatment of locally advanced or metastatic cSCC (11, 12). Very recently, neoadjuvant use of ICIs has been reported with a very favorable outcome (13). Here, we describe the case of a very advanced cSCC of the face involving the nasal and anterior oral cavities, treated with the anti-PD-1 antibody cemiplimab and hypofractionated radiation therapy.

## Case description

**Figure 1:**
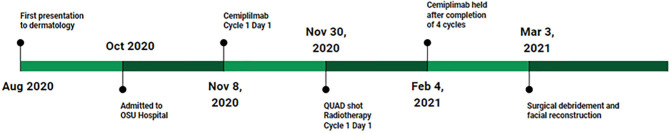
Case Timeline.

**Figure 2:**
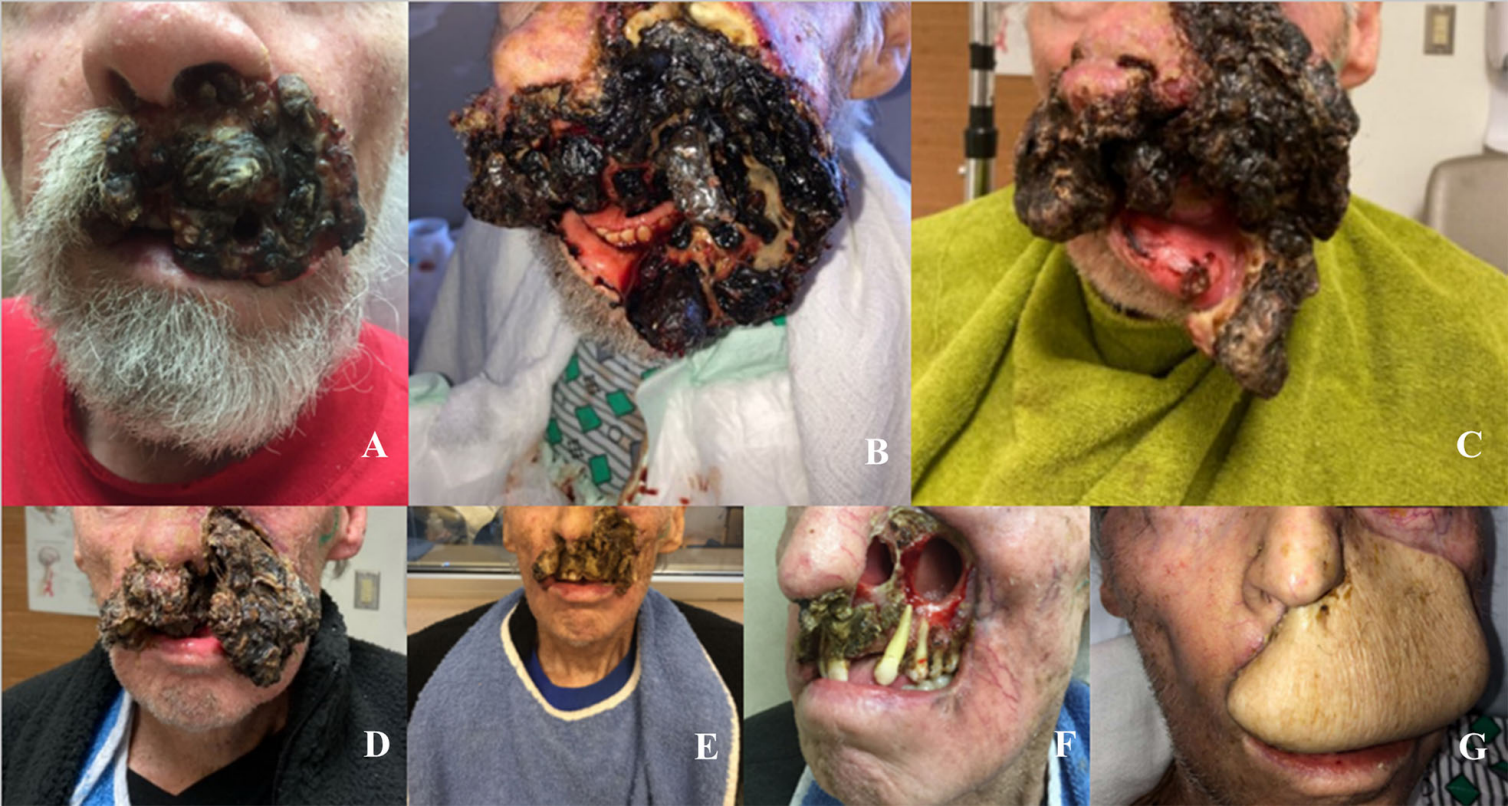
**(A-G)** Lesion During Treatment Progression: **(A)** August 2020, First presentation to dermatology; **(B)** November 6, 2020, prior to initiating cemiplimab; **(C)** December 4, 2020, after 1 cycle of cemiplimab; **(D)** December 22, 2020, after 2 cycles of cemiplimab and 1 cycle of QuadShot radiotherapy; **(E)** January 14, 2021, after 3 cycles of cemiplimab and 2 cycles of QuadShot radiotherapy; **(F)** February 11, 2021, after 4 cycles of cemiplimab and 2 cycles QuadShot radiotherapy; **(G)** October 2021, after debulking and reconstruction.

The patient is a man in his late 70s with a history of melanoma of the lower leg (1995), melanoma *in situ* (2012), and basal cell carcinoma of the forearm (1995). In 2017 the patient noticed a lesion on his left upper lip. The patient assumed the lesion was benign and neglected it. It grew gradually over the next 3 years, but in early 2020 enlarged suddenly and rapidly. He presented to the dermatology clinic in August 2020 (**Figures 1**, **2A**). At this point, his mass was exophytic and occupied the entire left upper lip with extension to the maxillofacial region. A shave biopsy indicated basal cell carcinoma with necrosis and a possible squamous cell carcinoma component. In mid-September 2020, the mass proliferated at a faster rate. A PET scan demonstrated 2 hyperactive cervical nodes with no evidence of metastatic disease distant to the neck. Genomics showed two pathogenic mutations, HRAS G13R and TP53C176F, and a variant of unknown significance, SMO A327T with a 50% allelic frequency, most likely confirming the basal-squamous cell nature of this cutaneous carcinoma. Immunohistochemistry was positive for PD-L1 with a combined positive score (CPS) of 111. In head and neck SCC, tumors with CPS scores ≥1 and ≥20 treated with pembrolizumab show improved overall survival (14). The initial plan was to directly admit the patient from the clinic the same day for palliative resection and further treatment; however, due to personal reasons, the patient decided against admission at that time.

## Diagnostic assessment

**Figure 3:**
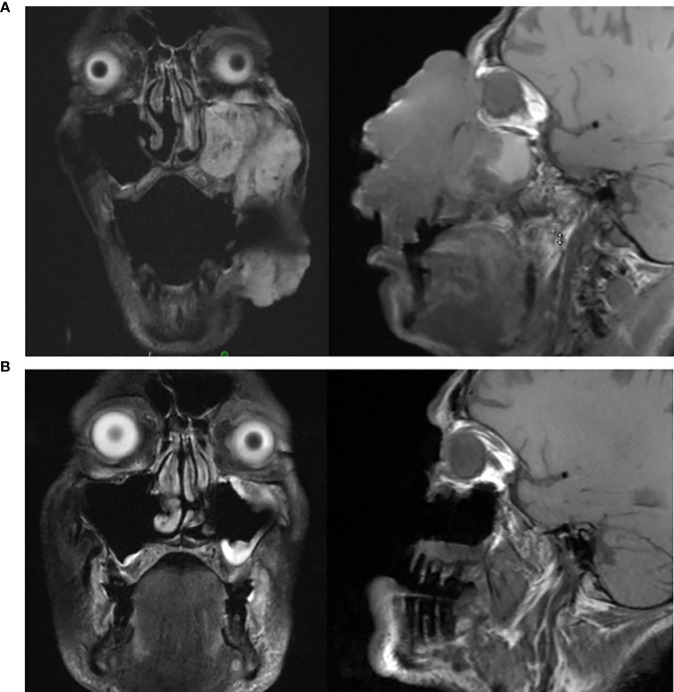
**(A, B)**: **(A)** (top): MRI of head prior to initiating cemiplimab; **(B)** (bottom): MRI of head following completion of cemiplimab.

In October 2020, the patient presented to plastic surgery. Unfortunately, his clinical status had declined with failure to thrive. His tumor burden had increased with progression further onto the left maxillofacial region with complete obliteration of the upper lip. TNM staging was T4a N2b M0, or stage IV. The patient was then admitted to inpatient medical oncology directly from the clinic. Once admitted, imaging revealed the destruction of the maxilla with the cancer extending into the left maxillary sinus and nasal cavity (**Figure 3A**). A left cheek full-thickness biopsy revealed poorly differentiated squamous cell carcinoma. The first reaction upon admission was to refer the patient to hospice, given the severity of the tumor burden (**Figure 2B**). However, the medical oncology consultant for skin cancers recommended a trial of neoadjuvant immunotherapy due to the potential for a rapid response and the otherwise ineluctable fate of the patient.

Cemiplimab was initiated on November 8, 2020. On November 23, 2020, the patient presented to the emergency department (ED) with an increase in the size of his facial mass and bleeding. This was assumed to be a flare (pseudoprogression) after immunotherapy. Radiation oncology recommended QUAD shot radiotherapy (RT) with 14 Gy, delivered in 4 fractions (twice per day for 2 days) every 3 weeks for 4 cycles. The lesion shrunk between the CT simulation and the radiation initiation and became drier. Cycle 1, day 1 QUAD shot was completed on November 30, 2020 (**Figure 2C**). Cycle 2 Day 2 cemiplimab was given on December 3, 2020. Following the second dose of cemiplimab + first radiation cycle, the lesion decreased by 75% with significant improvement from disease involvement near the eye. Cycle 2, day 1 QUAD shot was given on December 17, 2020 (**Figure 2D**), and cycle 3, day 1 cemiplimab was given on December 23, 2020. There was a further 75% reduction in the size of the mass (**Figure 2E**), but the patient developed grade-2 hypothyroidism. Radiation was then held after 50% of the dose was delivered. Following cycle 4 of cemiplimab, with cycle 4, day 1 on January 4, 2021, the patient developed two fistulas in his maxillary sinus and nasal cavity, which were determined to be sites of disease regression (**Figure 2F**). A facial MRI from January 27, 2021 revealed near-complete regression of the tumor in the inferior orbit (**Figure 3B**), indicating the patient may be able to avoid left orbital exenteration, which would have been indicated prior to ICI treatment. Cemiplimab was held on February 4, 2021 prior to cycle 5 of cemiplimab due to grade-1 transaminitis. The patient’s AST and ALT levels improved after treatment was held. Four biopsies were taken across the lesion on February 4, 2021, revealing no evidence of residual cancer. As his disease had significantly regressed, the care team felt that the patient had reached the point of maximal response. To salvage as much viable tissue as possible, the patient was referred to surgery for debridement of necrotic tissue and reconstruction (**Figure 2G**).

Post-surgical pathology examination revealed one persistent metastatic node with sarcomatoid differentiation and extracapsular extension only at nodal level 1. No other residual malignancy was identified at the surgical margins of the primary tumor. The patient had multiple complications post-surgery, including a refractory *C. difficile* colitis that affected his recovery. Twelve months postoperatively, there was no clinical or radiographic evidence of local recurrence. Further surgical interventions are planned in the next few months to debulk the facial flap and address periocular complications related to radiation and surgery.

## Discussion

Historically, local resection has been the primary intervention for cSCC. In this case, local resection was not a viable option due to extreme tumor burden, and the initial recommendation on admission was a referral to hospice care. Given advances in cancer treatments, with very effective therapies, it is paramount that physicians reflect carefully regarding referrals to hospice care, in the case of untreated advanced disease. Considerations for treatment should include the prognosis balanced with the potential benefits and risks of newer therapies. Through the administration of combination neoadjuvant cemiplimab and QUAD shot radiotherapy, there was a significant decrease in tumor burden, and subsequent biopsies surrounding the disease site found only necrotic tissue with no active disease. Immunotherapy and QUAD shot radiotherapy used independently are both effective, but there is scant data describing the treatment combination in the neoadjuvant setting (15). There is no data on the combination of radiotherapy and ICI for skin cancers. In other settings, clinical trials have shown mixed results. The Javelin 100 trial for locoregionally advanced head and neck squamous cell carcinoma was stopped early after a futility analysis (16). Landmark trials have shown a benefit of sequential immunotherapy after definitive chemoradiation in non-small-cell lung cancer (PACIFIC trial) (17) and esophageal cancer (CHECKMATE 577) (18). Multidisciplinary approaches utilizing combinations of systemic, radiation, and surgical treatment in locally advanced head and neck cancer improve patient outcomes (19, 20), but for cutaneous cancer further investigation is required into various combinations of techniques (19, 20), and multiple trials are currently ongoing. In this case report, the utilization of cemiplimab and radiation treatment caused dramatic regression of local disease without being able to tease out any specific benefit of each modality.

The recent developments involving treatment with PD-1 inhibitors and/or radiation therapy present new, promising alternatives. Cemiplimab was approved by the FDA for the treatment of locally advanced and metastatic cSCC in 2018 (21, 22) and achieved an overall response rate (ORR) of 41-50% (22–24). Both median progression-free survival (PFS) and overall survival (OS) were not reached. Reported 1-year OS rates ranged from 76-93% (22–24). The follow-up data from EMPOWER-CSCC-1 and phase 1 clinical trial NCT02383212 have shown a favorable side effect profile and long-lasting antitumor effects (25, 26). In a stage II trial involving neoadjuvant cemiplimab, there was an ORR of 30%, a complete pathologic response (CR) of 55%, and a major pathologic response (PR) of 15% of patients (27). These results were recently confirmed in another phase II trial with 79 patients enrolled of whom 54 had a clinical response, with 40 achieving a pathological complete response (13).

Hypofractionated radiation has been found to be effective in combination with anti-PD-1 therapy in the treatment of head and neck carcinoma (28–31). Neoadjuvant QUAD shot radiation has also been observed to downstage cancer of the oral cavity prior to definitive surgery in case series (32). While one study documented safety and response to QUAD shot with the addition of paclitaxel, a chemotherapy with radio-sensitizing properties (33), data on combination therapy is limited (34, 35). In this case, the tumor presented with bleeding, which supported the use of radiation to help obtain a better clinical result.

Initially, the shave biopsy indicated basal-squamous carcinoma, a potential weakness of this case. Alternatively, it is possible the tumor could represent basal cell carcinoma with marginal areas of G3 squamous degeneration. However, full-thickness biopsy favored poorly differentiated squamous cell carcinoma, and treatment was determined with cSCC as the final diagnosis.

Following completion of this combination therapy, all biopsied tissue surrounding the previous tumor site was clear of disease, except for one node with sarcomatoid degeneration that was resected. Reconstructive and salvage procedures were completed and tolerated. Without neoadjuvant treatment, surgery would have been substantially more extensive, requiring orbital exenteration and wider margins and would probably not have yielded an R0 resection. Combination therapies for difficult-to-treat cSCC disease may alleviate destructive surgeries in the future, much like the switch from radical mastectomies to lumpectomies for the treatment of breast cancer. Further research is needed to clarify the role of radiation in combination with cemiplimab in treating locally advanced unresectable cSCC. The bar has now been set quite high with a 51% complete pathological response seen in such patients with cutaneous squamous cell carcinoma (13).

## Data availability statement

The original contributions presented in the study are included in the article/supplementary material. Further inquiries can be directed to the corresponding author.

## Ethics statement

Written informed consent was obtained from the individual(s) for the publication of any potentially identifiable images or data included in this article.

## Author contributions

MB, MH, CV, and GT participated in acquisition and analysis of data. MB and MH wrote the first draft of the manuscript. CV, RW, and GT wrote sections of the manuscript. All authors contributed to manuscript revision, read, and approved the submitted version. All authors agree to be accountable for the accuracy and integrity of the manuscript.
